# Additive of cow dung weakened the influences of microbial interactions on nitrogen dynamic during composting of rice husks

**DOI:** 10.3389/fmicb.2025.1641502

**Published:** 2025-11-04

**Authors:** Bin Zhang, Delong Meng, Xichun Wang, Jin Hu, Jianqiang Fan, Xuan Li, Zhendong Yang, Wei He, Deying Zhou, Yiqiang Cheng, Jingjing Li, Junliang Zou, Zhenghua Liu

**Affiliations:** ^1^College of Chemistry and Bioengineering, Hunan University of Science and Engineering, Yongzhou, China; ^2^School of Minerals Processing and Bioengineering, Central South University, Changsha, China; ^3^Yongzhou Company of Hunan Tobacco Company, Yongzhou, China; ^4^Technology Center, Fujian Tobacco Industrial Corporation, Xiamen, China; ^5^College of Plant Protection, Hunan Agricultural University, Changsha, China; ^6^School of Architecture and Civil Engineering, Chengdu University, Chengdu, China; ^7^Forestry Development Department and Climate Centre, Teagasc, Wexford, Ireland

**Keywords:** composting, microbial network, biotransformation, seedling substrate, metagenome

## Abstract

Rice husk (RH) and cow dung (CD) are two of the most abundant agricultural solid waste. Converting these residues into peat-free substrates through co-composting supports sustainable agricultural development. A 40-day rice husk composting experiment was conducted to assess the effects of cow dung addition on microbial networks and carbon–nitrogen dynamics using 16S rRNA and metagenomic analyses. Furthermore, Furthermore, we prepared seedling substrates from composts of RH alone and RH combined with CD (RHCD), and evaluated their plant growth–promoting effects. The addition of cow dung (CD) to rice husk (RH) composting increased the average temperature from 52.8 °C to 60.1 °C and acted as a pH buffer, maintaining values around 7.4. CD significantly (*p* < 0.05) enhanced microbial network complexity, as indicated by larger network size and higher average degree, but disrupted the linear correlations between network properties and carbon or nitrate nitrogen contents (*p* > 0.05). This decoupling suggests that CD weakened the linkage between microbial interactions and carbon or nitrogen biotransformation processes. CD also significantly suppressed (*p* < 0.05) denitrification-related genes (*norB*, *nir* and *nar*) after the thermophilic phase, implying reduced nitrogen loss during compost maturation. We further found that larger network size or higher average degree reduced the abundance of key genes involved in assimilatory nitrite reduction (e.g., *nirBD*), while increasing those related to denitrification (e.g., *nirK* and *nirS*). Moreover, seedling substrates derived from RH (95.06%) and RHCD (93.21%) composts achieved higher germination rates of Solanaceae crops than the commercial peat-based substrate (81.48%). Germination rate and seedling biomass were positively correlated with dissolved organic carbon (*r* = 0.820, *p* = 0.045) and ammonium nitrogen (*r* = 0.858, *p* = 0.029), respectively. These findings advance the understanding of microbial interaction regulating carbon and nitrogen cycling during RH composting, and support the sustainable production of peat-free seedling substrates from agricultural waste.

## 1 Introduction

Proper disposal of agricultural solid waste has become a serious global concern, especially in agricultural countries, such as China, India and Indonesia (Pagliari et al., 2020; Singh et al., 2024). One of the most representative and abundant agricultural solid waste is rice husk (RH), with an annual global production of approximately 150 million tons (Kordi et al., 2024). China, as the biggest producer and processer of rice, has an annual production of 40 million tons of rice husk (Li et al., 2011). Reusing this unavoidable by-product is essential for the sustainability of rice production. In China, the traditional practice has been to burn RH and use the resulting ash improve soil properties, such as water retention and aeration, but this approach is no longer encouraged due to high CO₂ emissions. Alternatively, composting treatment is applied to transform rice husk into a seedling substrate, which exhibits excellent properties in terms of porosity, container capacity, and air space (Huang et al., 2019). However, the bioconversion rate of organic matter is remains low during composting of rice husk.

Co-composting rice husk with cow dung could further improve the conversion of organic matter. For example, cow dung introduces a large number of thermophiles, such as *Myceliophthora*, *Symbiobacterium* (Meng et al., 2019) and *Caldoanaerobacter subterraneus* (Yokoyama et al., 2007), which increase composting temperature, prolong the thermophilic phase, and accelerate the bioconversion of organic materials (Liu et al., 2011). Meanwhile, cow dung introduced more thermotolerant and thermophilic decomposers of cellulose, hemicellulose and lignocellulose during composting, such as Firmicutes and Actinobacteria (Zhang Z. et al., 2023). Collectively, these results indicate that composting performance is strongly governed by the composition and functional potential of microbial communities. Although the succession of microbial communities during the co-composting of rice husk and cow dung has been investigated (Duan et al., 2021), the microbial mechanisms underlying organic matter biotransformation remain unclear.

In this study, we applied composting approach to convert rice husks and cow dung into peat-free seedling substrates. We investigated the microbial mechanisms underlying carbon and nitrogen biotransformation, and assessed the plant growth-promoting potential of the resulting substrates and identified key factors contributing to their efficacy. To elucidate the underlying mechanisms, we investigated the biotransformation of carbon and nitrogen during composting of rice husk alone and in combination with cow dung using 16S rRNA gene sequencing and metagenomic analysis. We hypothesized that (i) the addition of cow dung reshapes microbial interaction network, and modulates carbon and nitrogen transformation processes, and (ii) the enrichment of available carbon and nitrogen in RH–CD compost mixtures would promote seed germination.

## 2 Materials and methods

### 2.1 Experiment setup and sample collection

The rice husk composting experiment was conducted in Yongzhou city, Hunan Province (26.4197° N, 111.6959° E) in May, 2024, with an average ambient temperature of 24 °C, an average altitude of 200 m, and an annual mean relative humidity of 79%. In the control group (RH), composting materials included 1,000 kg of crushed rice husk (filtered by a 3-mesh sieve), 5 kg of urea, 5 kg of corn starch, and 4 kg of fermentation aids (FJZ1201, Beijing China Healthead Science & Technology Co., Ltd.) that was microbial decomposer improving biotransformation of organic matter. In the treatment group (RHCD), 300 kg of cow dung was additionally incorporated into the composting mixture. The RH materials were mixed with municipal water to a moisture content of 60%, piled into a heap (10 m × 2 m × 1.5 m), and covered with plastic film. We determined these parameters based on the production practices of local enterprises. The carbon and nitrogen contents of the raw materials are presented in Supplementary Table S1, including rice husk, cow dung, urea, corn starch, and fermentation aids. Overall, the carbon nitrogen ratios of composting materials in RH and RHCD were 64.8 and 41.65, respectively.

The composting piles were manually turned every 10 days during the 30-day composting period, resulting in three turnings on days 10, 20, and 30. This schedule ensured adequate aeration and uniform decomposition. When the moisture content of the compost piles fell below 60%, municipal water was added to adjust it to 65–70%. Eight replicate samples were collected at each of the five composting phases, including the heating, thermophilic, cooling, and mature phases. Samples were taken at a depth of 0.4 m, with 150–200 g collected per replicate, for physicochemical and microbial analyses.

### 2.2 Physicochemical analysis

We measured the temperature of the composting pile at a depth of 0.4 m every day. Physicochemical analysis of composting samples was conducted according to standard methods for soil physical and chemical analysis (Liu et al., 1996). The pH of the compost suspensions, prepared at a solid-to-water ratio of 1:2.5 (w/v), was measured using a pH meter (PHS-3C, Lei-ci, China). The total carbon content was determined by the dry combustion method, and dissolved organic carbon (DOC) was measured using the K₂Cr₂O₇ volumetric method with external heating. Total nitrogen (TN) was quantified using the semimicro Kjeldahl method. Nitrate nitrogen (NO_3_^−^-N) and ammonium nitrogen (NH₄^+^-N) were extracted with 25 mL of potassium chloride from 2.5 g of compost sample, and their concentrations were determined using a continuous flow analyzer (San++, Skalar, Holland).

### 2.3 Evaluating seedling effects

We prepared three types of seedling substrates and evaluated their effects on tobacco seedling growth: (i) a rice husk–based compost substrate (RH), (ii) a mixed compost substrate containing rice husk and cow dung (RHCD), and (iii) a conventional commercial peat-based seedling substrate (PSS). The compositions of these substrates are presented in Supplementary Table S2.

Tobacco seedlings were cultivated in the three substrate treatments using a floating-tray seedling system. During the seedling stage, the emergence rate was recorded for each treatment. At 30 days after sowing, five seedlings from each group were randomly sampled to measure agronomic traits, including the number of leaves and roots, shoot biomass, root biomass, and root surface area.

### 2.4 Amplicon sequencing and sequence processing

The HiPure Soil DNA Mini Kit was used to extract microbial DNA, and the DNA quality was evaluated using a NanoDrop One spectrophotometer (NanoDrop Technologies, Wilmington, DE) and a Qubit 3.0 Fluorometer (Life Technologies, Carlsbad, CA, USA). A pair of primers, 341F (5′-CCTACGGGNGGCWGCAG-3′) and 805R (5′-GACTACHVGGGTATCTAATCC-3′), was used to amplify the V3-V4 region of the 16S rRNA gene of the microbial community. The polymerase chain reaction (PCR) mixture contained 2 μL each of the forward and reverse primers, 3 μL of template DNA, and 25 μL of VAHTS HiFi Universal Amplification Mix, with nuclease-free water added to a final volume of 50 μL. The amplification program was set as follows: (i) initial denaturation at 98 °C for 45 s; (2) denaturation at 98 °C for 15 s, annealing at 60 °C for 30 s and extension at 72 °C for 50 s, repeated for 25 cycles; (3) final extension at 72 °C for 10 min and hold at 4 °C. The sequencing library was constructed using the VAHTS® Universal DNA Library Prep Kit (Illumina) following the manufacturer’s protocol. After quality inspection, sequencing was conducted on the Illumina NovaSeq6000 platform (Illumina, Inc., CA, USA).

Sequence processing was conducted using QIIME 2 software (Hall and Beiko, 2018). Briefly, sequences with low quality were filtered based on Phred+33 quality parameters and the remaining sequences were used to generate the amplicon sequence variants (ASVs) using the DADA2 plugin. The representative ASV sequences were taxonomically classified using the RDP classifier against the SILVA database (v138) (Quast et al., 2013).

### 2.5 Metagenomic sequencing and nitrogen metabolism analyses

Microbial DNA was extracted using the DNeasy® PowerSoil® Kit according to the manufacturer’s instructions for metagenomic sequencing. Sequencing libraries were constructed with the VAHTS® Universal Plus DNA Library Prep Kit (Illumina, USA) following the supplier’s protocol. DNA quality was assessed using a NanoDrop One spectrophotometer (NanoDrop Technologies, Wilmington, DE, USA) and a Qubit 4.0 Fluorometer (Life Technologies, Carlsbad, CA, USA). Metagenomic sequencing was performed on the Illumina HiSeq PE250 platform (Illumina, Inc., CA, USA). Low-quality reads were filtered based on Phred+33 quality scores using the FASTX-Toolkit (v0.0.14) (Gordon and Hannon, 2017). Contig assembly of the remaining sequences was performed by MEGAHIT (v1.2.9) software (Li et al., 2015) with default parameters. Genes in contig sequences were annotated by Prokka (v1.14.6) software (Seemann, 2014). Functional genes associated with nitrogen metabolism were further identified using Diamond (v0.8.22.84) (Buchfink et al., 2015) against the NCyc database (Tu et al., 2019) with default parameters.

### 2.6 Network analyses

To construct the microbial network, correlations between ASV pairs were estimated using the SparCC method (Kurtz et al., 2015). ASVs with relative abundances below 0.0002% were filtered out, and SparCC correlations were calculated for the remaining ASV pairs. Only significant correlations with *p* < 0.05 were retained, and the correlation threshold was determined by random matrix theory (RMT) (Deng et al., 2012). Subnetworks for each sample were extracted from the global microbial network based on the ASVs present. Network properties were calculated using the “cal_network_attr” function of the microeco package, including average degree, average path length, network diameter, clustering coefficient, density, heterogeneity, and centralization. Network nodes were classified into four categories based on within-module connectivity (Zi) and among-module connectivity (Pi) (Guimera and Nunes Amaral, 2005): (i) module hubs with Zi > 2.5 and Pi < 0.62; (ii) connectors with Zi < 2.5 and Pi > 0.62; (iii) network hubs with Zi > 2.5 and Pi > 0.62 and (iv) peripheral nodes with Zi ≤ 2.5 and Pi ≤ 0.62. All network analyses were performed using the “microeco”package (v1.11.0) (Liu et al., 2021).

### 2.7 Statistical analyses

Linear regression analysis was used to estimate the relationships between network properties and environmental factors using the “lm” function (Chambers and Hastie, 1992). Venn diagram analysis was conducted to detect the difference in species pool of network roles, which was performed with “ggvenn” package (v0.1.9) (Yan, 2021). The mantel test was used to calculate the association between network roles and environmental factors, which was implemented within “linkET” package (v0.0.7.4)^1^. Random forest analysis (Breiman, 2001) was used to identify the most important genes involved in nitrogen metabolism for TN, NO₃^−^-N and NH₄^+^-N. Partial least squares path modeling (PLSPM) analysis was used to explore the contribution of microbial network, nitrogen metabolism and carbon pool to nitrogen transformation, which was brought out by “plspm” package (v0.5.1) (Sanchez et al., 2013). Visualization of statistical analysis was conducted by “ggplot2” package (v3.5.1) (Wickham, 2016). All statistical analyses were performed in the R (v4.1.1) environment (Team, R. C., 2000).

## 3 Results

### 3.1 Carbon and nitrogen dynamics throughout the composting process

**Figure 1:**
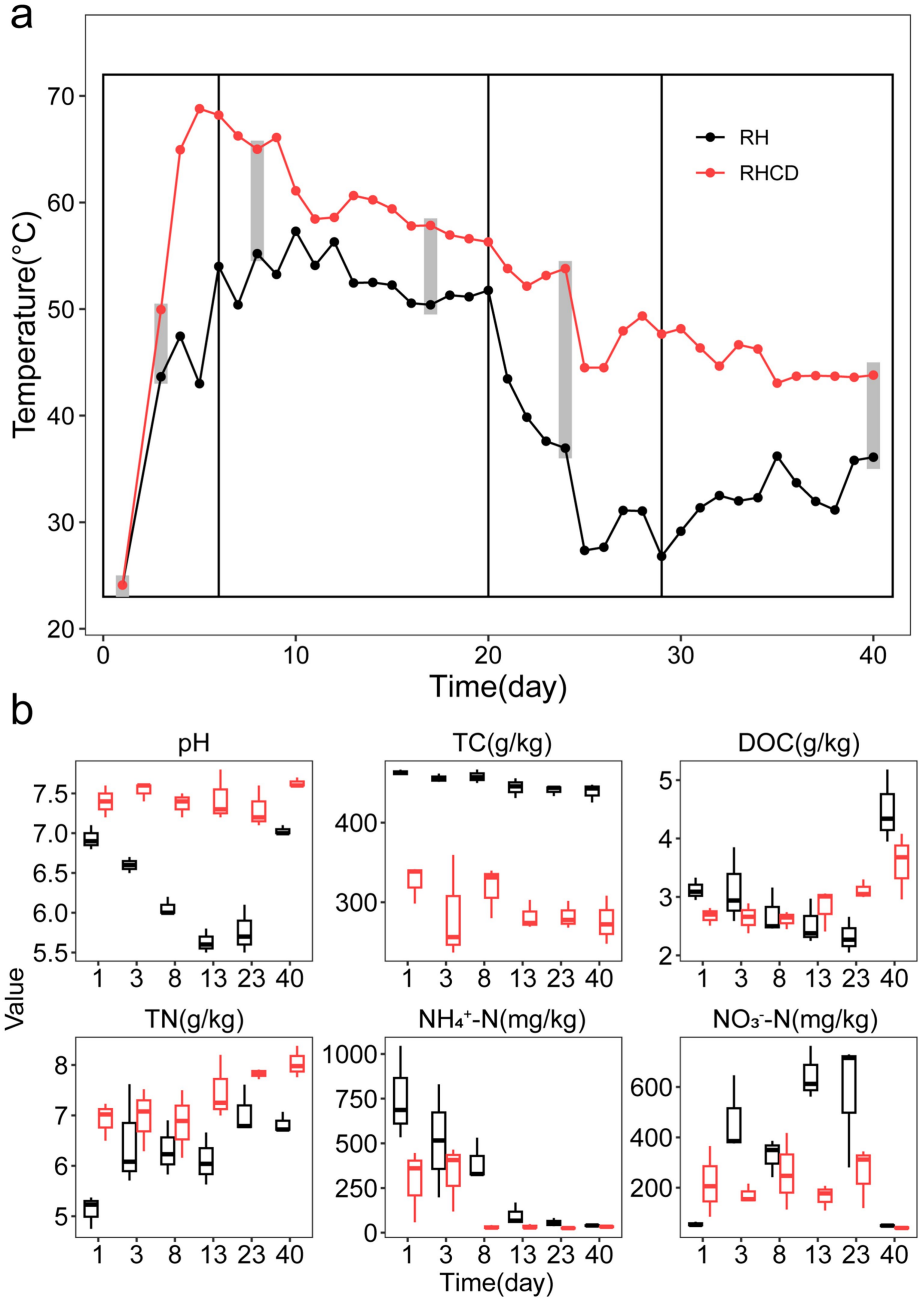
Temperature **(a)** and physicochemical factors **(b)** during composting process. TC: Total carbon content. DOC: Dissolved organic carbon. TN: Total nitrogen. NH_4_^+^-N: Ammonium nitrogen. NO_3_^−^-N: Nitrate nitrogen.

As shown in Figure 1a, we divided the 40-day composting experiment into four phases based on temperature dynamics, including heating phase (0–6 d), thermophilic phase (7–20 d), cooling phase (21–29 d) and mature phase (30–40 d). Throughout the composting process, temperature in the rice husk with cow dung (RHCD) treatment was on average 11 °C higher than that in rice husk (RH) treatment (paired t-test, *p* < 0.001). Specifically, at the thermophilic phase, the average temperature in RHCD was 60.1 (±3.4) °C, while that of RH was 52.8 (±2.2) °C. During the cooling phase, average temperature of RHCD and RH was 49.1 (±3.7) °C and 32.3 (±5.2) °C, respectively. During the mature phase, average temperature of RHCD and RH were 44.9 (±1.7) °C and 32.9 (±2.3) °C, respectively. Furthermore, the pH of RHCD remained stable at 7.4 (±0.2), whereas that in RH decreased from 6.9 to 5.6 at the cooling phase and then increased to 7.0 at the mature phase (Figure 1b).

As composting progressed, the total carbon content (TC) in RH was significantly higher than that of RHCD across all four phases (*p* < 0.001; Figure 1b). Specifically, TC in RH gradually decreased from 462.78 (±3.44) g/kg to 438.6 (±11.74) g/kg (5.2% reduction; *p* = 0.001), while TC of RHCD decreased from 326.20 (±24.13) g/kg to 276.11 (±30.25) g/kg (15.4%; *p* = 0.043). Dissolved organic carbon (DOC) in RHCD significantly increased from 2.67 (±0.15) g/kg to 3.57 (±0.57) g/kg (33.7%., *p* = 0.05). In contrast, DOC in RH decreased from 3.12 (±0.19) g/kg to 2.33 (±0.31) g/kg during cooling phase (25.3%, *p* = 0.013), before sharply increasing to 4.49 (±0.63) g/kg at the mature phase (92.7%, *p* = 0.027; Figure 1b). These results suggested that the addition of cow dung significantly altered carbon dynamics throughout the composting process.

We also found that the total nitrogen content (TN) in RHCD was significantly higher than that in RH across all four phases (*p* < 0.001; Figure 1b). Specifically, TN in RHCD significantly increased from 6.92 (±0.38) mg/kg to 8.04 (±0.31) mg/kg (16.2%, *p* = 0.003), while that in RH group significantly increased from 5.12 (±0.32) mg/kg to 6.83 (±0.21) mg/kg (33.4%, *p* < 0.01). Conversely, ammonium nitrogen (NH₄^+^-N) in RHCD and RH was 288.12 (±203.59) mg/kg and 755.30 (±262.41) mg/kg at the initial stage, and rapidly declined to 32.93 (±3.94) mg/kg (88.6%, *p* < 0.01) and 39.87 (±3.87) mg/kg (94.7%, *p* < 0.01) at the thermophilic phase, respectively. Interestingly, nitrate nitrogen (NO₃^−^-N) of RHCD maintained at a range of 164.69 mg/kg to 259.32 mg/kg at the first three phases, before decreasing sharply to 40.56 (± 4.18) mg/kg by 75.4% (*p* < 0.05) at the mature phase. A similar trend was observed in RH, where initial NO₃^−^–N levels were higher (325.90–645.96 mg/kg) and decreased to 48.80 (± 3.44) mg/kg by 85.0% (*p* < 0.05). These results showed that addition of cow dung reduced nitrogen loss in the forms of NH₄^+^-N and NO_3_^−^-N during composting of rice husk.

### 3.2 Effects of microbial interaction network on carbon and nitrogen dynamic

**Figure 2:**
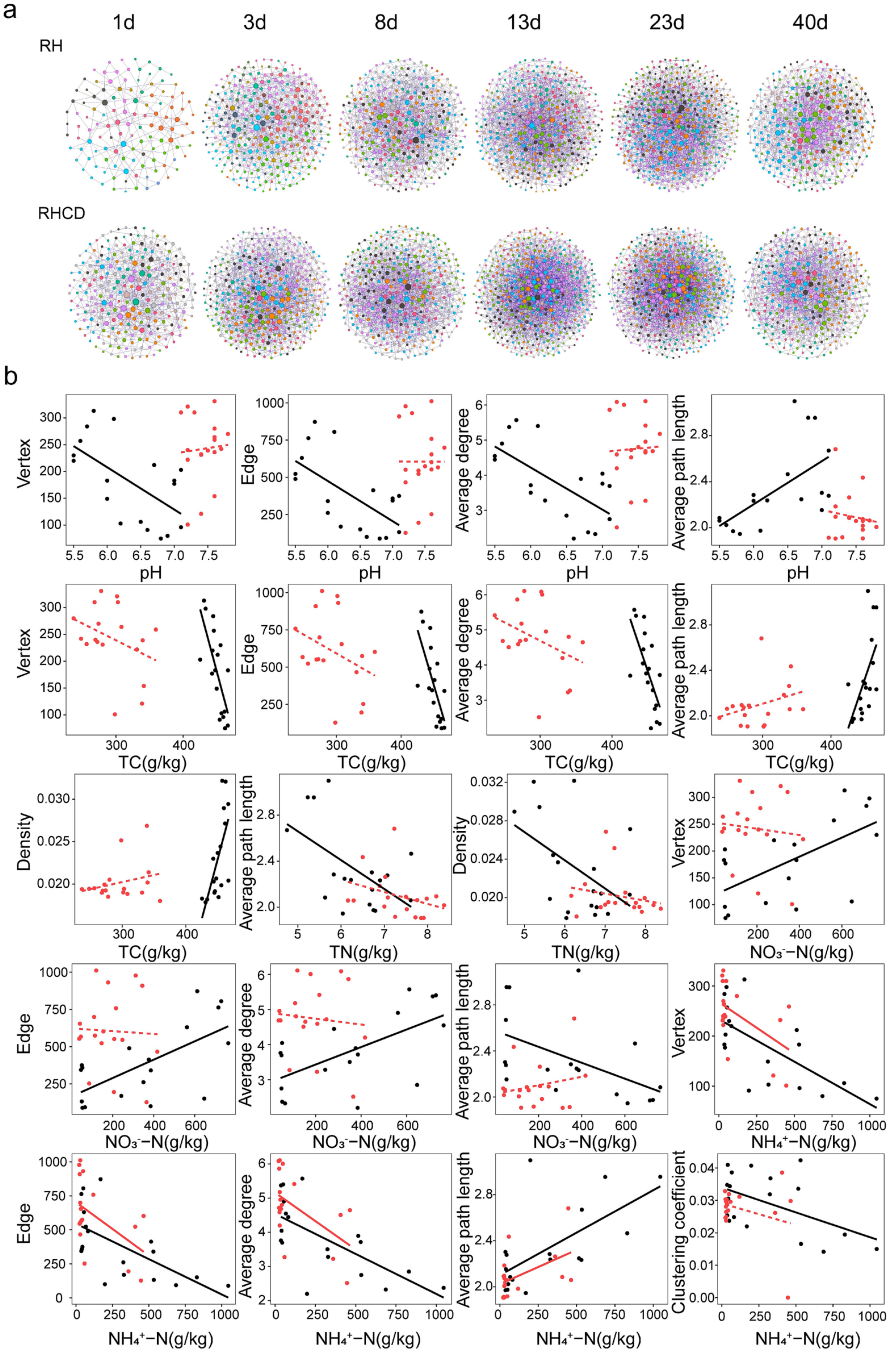
Succession pattern in microbial network during composting process **(a)**, and correlations between network properties and environmental features **(b)**. Black: RH. Red: RHCD.

Our results showed that the microbial network size of the RHCD was significantly larger than that of RH during the heating and mature phases (Figure 2a). For example, the microbial networks of RHCD contained 251 and 245 vertices at the heating and mature phases, respectively, compared with 132 and 189 vertices in RH at the same phases. However, the microbial networks of RHCD exhibited lower complexity than those of RH during the last three phases (Figure 2a). For instance, average clustering coefficients of microbial network in RHCD were 0.031, 0.024, 0.026 and 0.026 at 8d, 13d, 23d and 40d, respectively, whereas those of RH were 0.048, 0.032, 0.036 and 0.040. Consistently, the average network density of RHCD was 0.021 across the thermophilic, cooling and mature phase, which was significantly lower than that of RH (0.024; paired *t*-test, *p* < 0.001). Similarly, network centralization of RHCD group was 0.021 (±0.002) and significantly lower than that of RH (0.024 ± 0.002) at the thermophilic phase (*p* = 0.006). In both of RHCD and RH, during the first three phase, the average degree increased significantly, while the average path length and network diameter decreased (all *p* < 0.05). In contrast, during the mature phase, the average degree decreased, while the average path length increased (all *p* < 0.05). Moreover, network heterogeneity in both RHCD and RH remained similar and showed no significant changes throughout the composting process (all *p* > 0.05).

The associations between microbial network properties and carbon or nitrogen dynamics were generally weaker in RHCD than in RH (Figure 2b and Supplementary Table S3). In RH, pH and TC were both negatively correlated with network size (number of vertices and edges) and average degree, but positively correlated with network density (all *p* < 0.05). TN showed negative correlations with average path length and positive correlations with the clustering coefficient (*p* < 0.05), suggesting that higher TN may promote node aggregation. NO_3_^−^-N was positively correlated with network size and average degree but negatively correlated with density (*p* < 0.05). In contrast, these correlations became insignificant in RHCD. Consistently, in both treatments, NH_4_^+^-N was negatively correlated with network size and average degree but positively correlated with average path length and density (all *p* < 0.05). Overall, cow dung addition weakened the coupling between microbial network structure and carbon or nitrogen dynamics.

### 3.3 Influences of keystone species on carbon and nitrogen dynamic

**Figure 3:**
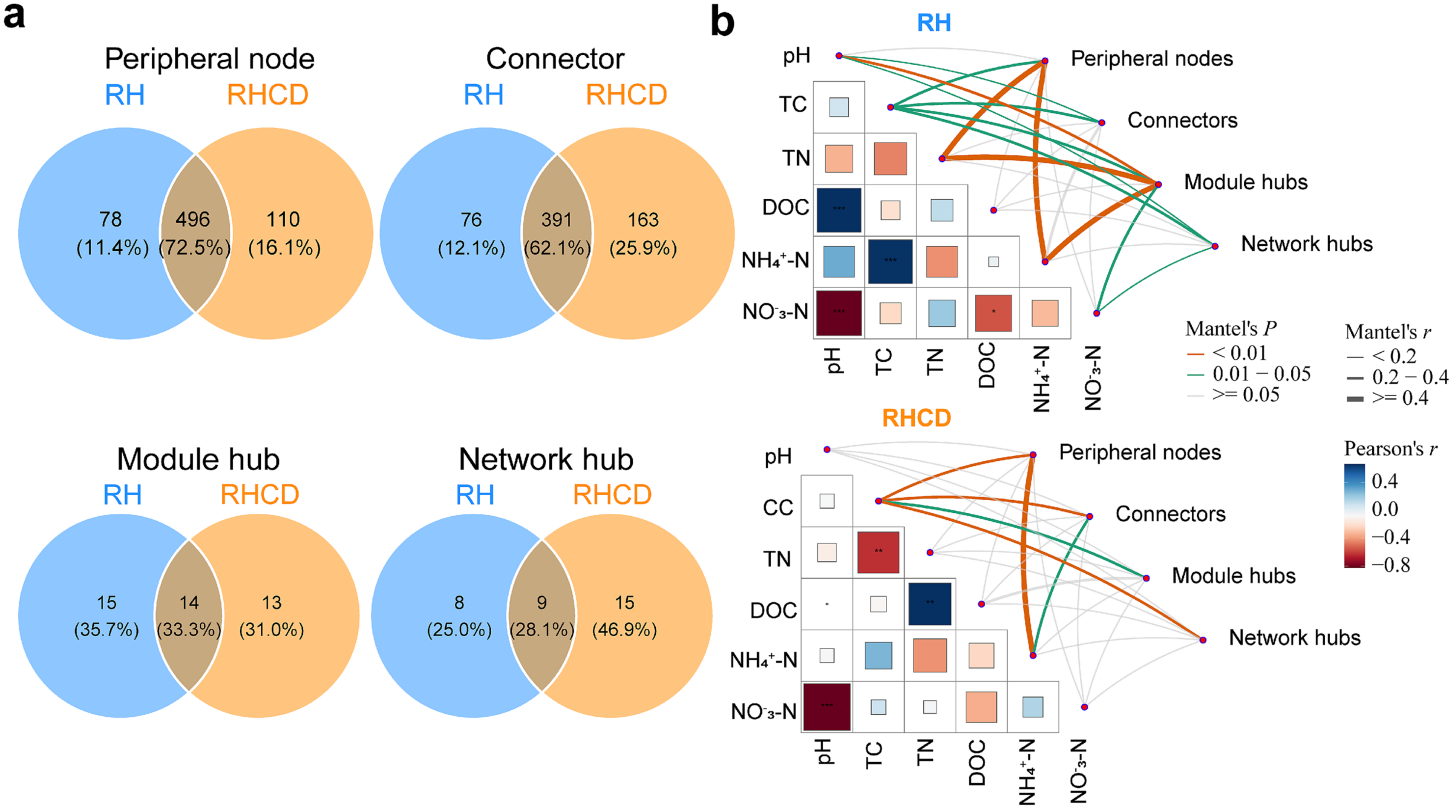
Differences in species pool of network topological roles between RH and RHCD groups **(a)** and links of network topological roles to environmental features **(b)**.

Our results showed that the number of network connectors in the RHCD was significantly higher than in the RH group during the heating, cooling, and mature phases (all *p* < 0.05; Supplementary Figure S1). However, there were no significant differences in the number of module hubs or network hubs throughout the composting process. Notably, the sizes of the species pools in connectors and network hubs of RHCD (76 and 15, respectively) were larger than those of RH (76 and 8; Figure 3a). In contrast, the species pool of module hubs in RHCD (27) was slightly smaller than in RH (29). Moreover, there were obvious differences in the compositions of these three species pools between RHCD and RH (Supplementary Figure S2). For connectors, compared with RH, specific candidates in RHCD included *Longimicrobia*, BD7-11 and *Methanomicrobia*. *Longimicrobia* persisted throughout the heating, thermophilic, and mature phases; BD7-11 appeared during the thermophilic and cooling phases; and *Methanomicrobia* was detected from the thermophilic to the mature phase. For module hubs, unique taxa in RHCD included subgroup 6 of *Acidobacteria* and *Anaerolineae*, which appeared during the heating–cooling and cooling–mature phases, respectively. In RHCD, the network hubs included *Rhodothermia* and *Anaerolineae*, detected during the heating–thermophilic–cooling and heating–cooling phases, respectively. *Thermoleophilia* acted as a connector in both treatments, spanning the heating to cooling phases in RHCD but appearing only during the cooling phase in RH.

Furthermore, we found that the four topological roles had distinct effects on carbon and nitrogen dynamics (Figure 3b). Specifically, for peripheral nodes, they were significantly associated with TC, TN and NH₄^+^-N in RH, while they were associated with TC and NH₄^+^-N in RHCD. For connectors, they were significantly correlated with pH and TC in RH, while that was TC and NH₄^+^-N in RHCD. For module hubs, they were significantly related to pH, TC, TN and NH₄^+^-N in RH, while they were only associated with TC in RHCD. For network hubs, they significantly affected pH, TC and NO_3_^−^-N in RH, while they were only correlated with TC in RHCD.

### 3.4 Changes in nitrogen metabolism during composting process

**Figure 4:**
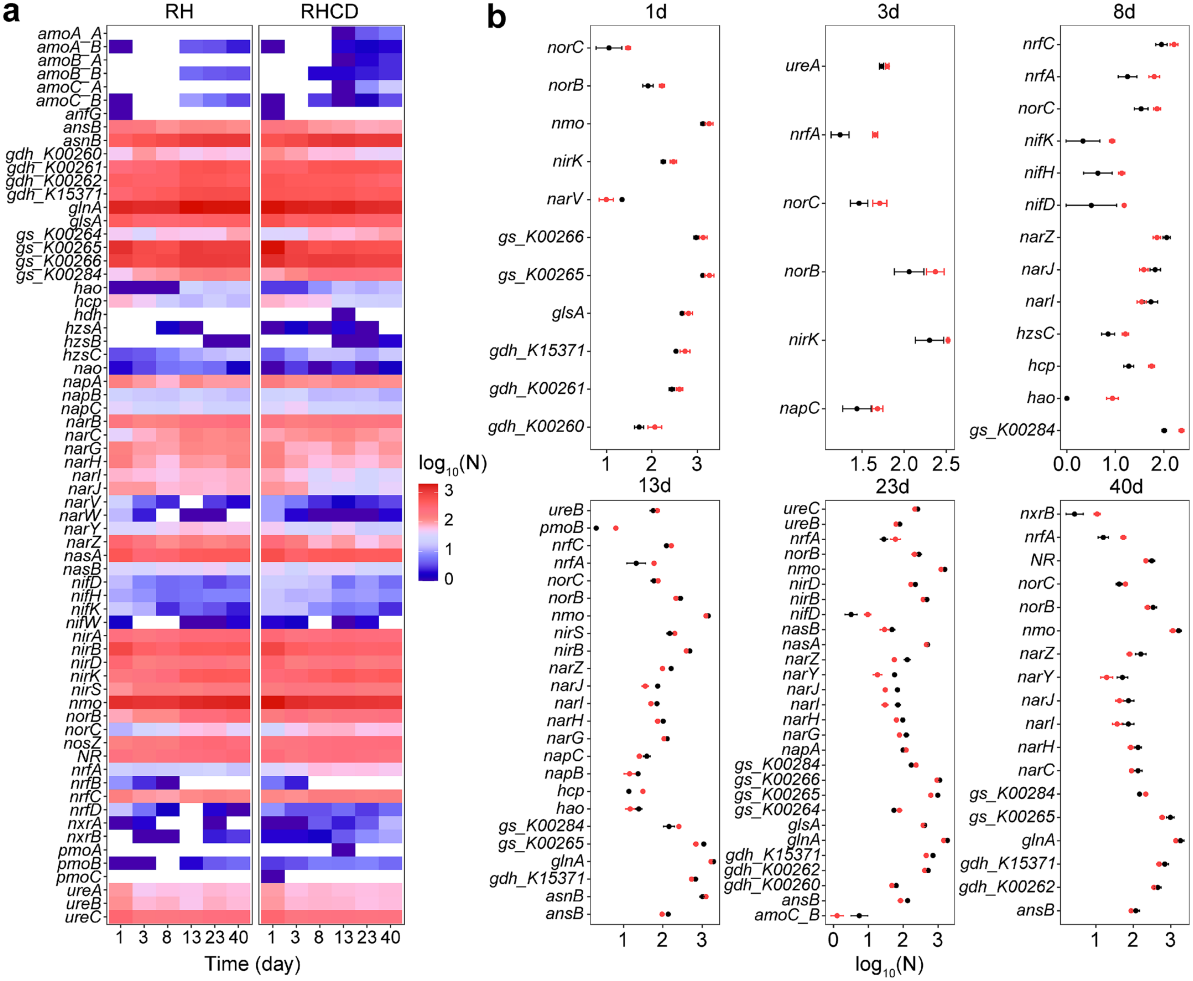
Changes in genes involving in nitrogen metabolism **(a)** and their differences **(b)** during composting process. Black: RH. Red: RHCD.

Our results showed that addition of cow dung significant changed nitrogen metabolism of microbial community during rice husk composting, which may reduce nitrogen loss after thermophilic phase. For example, genes encoding archaeal ammonia monooxygenase (NH_4_^+^ → NO_2_^−^) were detected only in RHCD during the thermophilic, cooling, and mature phases (Figure 4a), including *amoA_A*, *amoB_A*, and *amoC_A*. At early stage of thermophilic phase (8 d), abundance of *nifDHK* encoding nitrogenase (N_2_ → NH_4_^+^) in RHCD became higher than that in RH, suggesting cow dung may enhance nitrogen input. After thermophilic phase (13 d), abundance of key genes associated with denitrification in RHCD fall below than that in RH, such as *narGHI* encoding respiratory nitrate reductase (NO_3_^−^ → NO_2_^−^) and *norB* encoding nitric oxide reductase (NO → N_2_O) (Figure 4b). At mature phase (40 d), abundance of *nxrB* encoding nitrite oxidoreductase (NO_2_^−^ → NO_3_^−^) in RHCD was higher than that in RH (Figure 4b), implying that cow dung may promote nitrogen retention in nitrate nitrogen. Additionally, after thermophilic phase (13 d), abundance of *nrfA* encoding cytochrome c nitrite reductase (NO_2_^−^ → NH_4_^+^) in RHCD was also higher than that in RH (Figure 4b), which facilitates nitrogen retention in ammonium nitrogen (Wang C. et al., 2024).

### 3.5 The contribution of key factors to nitrogen pool

**Figure 5:**
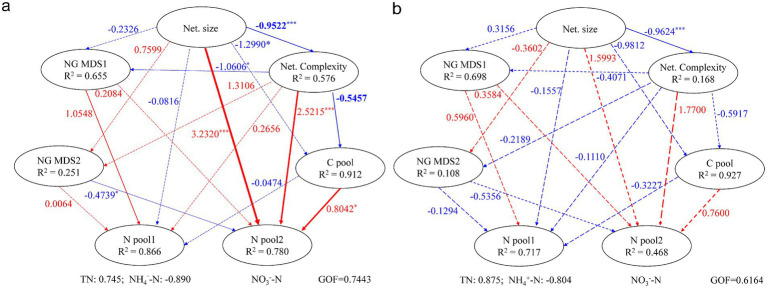
Partial least squares path model for influences of key factors on nitrogen dynamic in the RH **(a)** and RHCD group **(b)**. The line width represents the coefficient magnitude of model. The line colors represent the positive (red) or negative (blue) effects.

Results from partial least squares path modeling (PLSPM) revealed that cow dung addition reshaped the pathways contributing to nitrogen pool dynamics during composting (Figure 5 and Supplementary Table S4). In RH, nitrogen pool 1 loaded with TN and NH_4_^+^-N, was directly and significantly (*p* < 0.05) influenced by the first NMDS axis of nitrogen genes (NG NMDS1). Meanwhile, nitrogen pool 2 loaded with NO₃^−^-N, was significantly affected by NG NMDS2 (−0.4739), network size (3.232), network complexity (2.5215), and the carbon pool (0.8042) loaded with TC and DOC. In contrast, no variable exerted a significant direct effect on either nitrogen pool in RHCD. Network size mainly affected both nitrogen pools indirectly in RH (pool 1: −0.65; pool 2: −1.05), whereas these effects were weaker in RHCD (pool 1: −0.44; pool 2: −0.47). Consistently, the indirect effect of the carbon pool on nitrogen pool 1 was 2.386 in RH, stronger than that in RHCD (1.768; Supplementary Table S4).

Specifically, as shown in Supplementary Figure S3, the abundance of key genes encoding the two nitrate reduction systems, NarGHIJ and NarZYW, was suppressed by increases in temperature, the number of vertices and edges, average degree and clustering coefficient, but stimulated by shorter average path lengths. Moreover, increases in temperature, number of vertices and edges, and average degree decreased the abundance of key genes related to assimilatory nitrite reduction (*nirBD*; NO₂^−^ → NH_3_/NH₄^+^), but increased the abundance of genes associated with denitrification of nitrite (*nirKS*, *norBC*, *nosZ*; NO₂^−^ → N₂). Conversely, increases in average path length enhanced the abundance of genes involved in assimilatory nitrite reduction but reduced those related to nitrite denitrification. Additionally, increases in carbon content stimulated the abundance of genes related to nitrate reduction (*narGIJ* and *narYZ*) but suppressed those involved in nitrite denitrification (*nirKS* and *norC*).

### 3.6 Plant growth-promoting effects of composting products

Our results showed that composting products had stronger plant growth-promoting effects than the peat-based seedling substrate (PSS; Table 1). For instance, the germination rates of Solanaceae crops in the RH and RHCD composting products were 95.06% (±2.47%) and 93.21 (±1.23%), respectively, both significantly higher than that of PSS (81.48 ± 8.71%; all *p* < 0.05). Meanwhile, composting products generally enhanced root development. Specifically, the number of roots in RH (658 ± 125) and RHCD (616 ± 59) was slightly higher than in PSS (574 ± 141). The total fresh root biomass in composting products (RH: 0.77 g ± 0.22 g; RHCD: 0.70 g ± 0.11 g) was also higher than that in PSS (0.58 g ± 0.08 g). In contrast, the aboveground biomass of composting products (RH: 3.63 g ± 0.55 g; RHCD: 3.29 g ± 0.21 g) was lower than that of PSS (4.02 g ± 0.18 g). Similarly, the root surface area of RH (35.67cm^2^ ± 1.42 cm^2^) and RHCD (36.93 cm^2^ ± 5.11 cm^2^) was on average lower than that of PSS (42.67 cm^2^ ± 7.60 cm^2^).

**Table 1:** Influences of three seedling substrates on crop growth.

Group	Emergence rate (%)	Number of blades	Number of roots	Aboveground weight (g)	Root weight (g)	Root surface area (cm^2^)
RH	95.06 ± 2.47a	6.27 ± 0.23b	658.4 ± 125.20a	3.63 ± 0.55ab	0.77 ± 0.22a	35.67 ± 1.42a
RHCD	93.21 ± 1.23a	6.47 ± 0.23a	616.0 ± 59.49a	3.29 ± 0.21b	0.70 ± 0.11a	36.93 ± 5.11a
PSS	81.48 ± 8.71b	7.20 ± 0.40a	574.6 ± 141.06a	4.02 ± 0.18a	0.58 ± 0.08a	42.67 ± 7.60a

RH: Composting of rice husk. RHCD: Composting of rice husk with cow dung. PSS: Peat-based seedling substrate. The significance level of different letters is 0.05.

**Figure 6:**
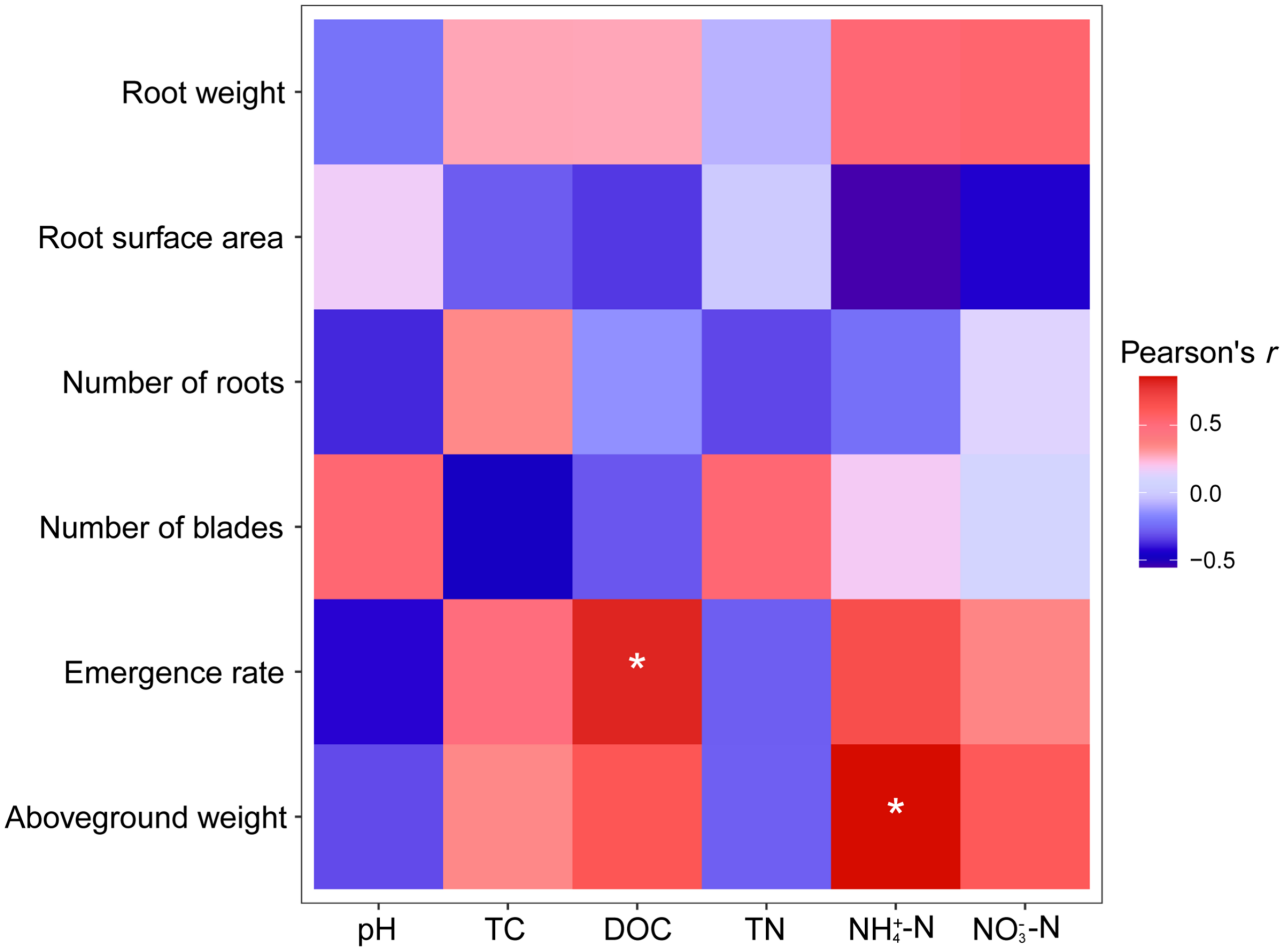
Correlations between seedling growth features and environmental factors. *p < 0.05.

Correlation analysis (Figure 6) showed that DOC in composting products was significantly and positively correlated with germination rates of Solanaceae crops (Pearson’s *r* = 0.820, *p* = 0.045). The concentration of ammonium nitrogen in composting products was also positively correlated with total biomass (*r* = 0.858, *p* = 0.029) and aboveground biomass (*r* = 0.862, *p* = 0.027).

## 4 Discussion

### 4.1 Addition of cow dung improve composting product quality of rice husk as seedling substrate

Addition of cow dung accelerated the heating rate, increased average temperature and extended thermophilic phase during composting, which may be contributed by diverse thermophilic microorganisms introduced from cow dung. For example, RHCD enriched *Thermoleophilia* that is typically thermophilic (Hu et al., 2019) and plays critical roles in bioconversion of organic maters (Chen et al., 2014). Similar results were observed in composting of cow dung and straw, which lead to a rapid increase of temperature during the heating phase (Liu et al., 2011), and promotes the partial depolymerization of lignocellulosic biomass (López et al., 2021), such as rice straw (Tang et al., 2007) and wheat straw wastes (Fan et al., 2006). We also found that DOC in RHCD showed a gradual accumulation. With elevated temperatures, microorganisms originating from cow dung exhibit higher activity in degrading cellulose, hemicellulose, and lignin into soluble monosaccharides or organic acids. Consequently, TC in RHCD decreased by 15.4% within 40 days, showing a greater reduction than that in RH (5.2%), but which means that cow dung may cause carbon loss.

Cow dung serving as pH buffer improves quality of composting products. We found that co-composting rice husk and cow dung showed a stable neutral pH at 7.4, which was lower than that of cow dung composting (8.07–8.86) (Wang et al., 2017) and higher than that of rice husk composting. This may be due to the neutralization of dissolve organic acids from rice husk decomposition and NH_3_ from hydrolysis of protein and urea in cow dung (Anda et al., 2008; Ba et al., 2020). It indicated that cow dung presented a pH buffer maintaining neutral pH, which favors nutrient solubility and uptake (Barrow and Hartemink, 2023). Meanwhile, such buffering capacity reduces the risk of ammonia toxicity, and provides a more consistent microenvironment for seed germination and root establishment (Shoemaker and Carlson, 1990).

Addition of cow dung promote stable accumulation of DOC. This may be because microbial community from cow dung have high activity in degrading cellulose, hemicellulose and lignin to soluble monosaccharides or organic acids, which can continuously promote the accumulation of DOC. Consequently, TC in RHCD decreased by 15.4% within 40 days, showing a greater reduction than that in RH (5.2%), but which means that cow dung may cause carbon loss. However, DOC in RH decreased before the mature phase and then sharply increased. This may be because the low microbial activity in degrading rice husk was insufficient to generate enough DOC to sustain microbial growth before and during the cooling phase. At manure phase, abundance of decomposers of rice husk increased, such as *Thermopolyspora* (Supplementary Figure S4) that efficiently degrade cellulose and hemicellulose (Chen et al., 2025), stimulating the rapid accumulation of DOC. In comparison, stable DOC accumulation in RHCD could facilitate better monitoring and control of the composting process.

### 4.2 Microbial complex interactions drive nitrogen transformation

Nitrogen dynamic is strongly influenced by microbial interactions during composting (Wang et al., 2022; Wang S. et al., 2024). For example, nitrogen conversion in chicken manure composting were related to network complexity of bacterial communities (Li et al., 2022). Consistently, we found that the network clustering coefficient was positively correlated with TN in RH. Network clustering coefficient describes the modular structure and the tendency to form highly connected clusters in microbial networks. Higher clustering coefficients may reflect the tendency of nitrifiers and denitrifiers to form cooperative guilds, which can balance nitrification and denitrification processes and thereby promote total nitrogen accumulation (Liu et al., 2024). It was consistent that diverse core species clustering together into modules can enhance organic nitrogen transformation and regulate compost maturity in cow-dung-driven composting (Liu et al., 2024). We also found that average path length had negative influences on total nitrogen, but that was positive on NH₄^+^-N. Shorter path length between two species in microbial co-occurrence network means that they could interact or cooperate more efficiently (Deng et al., 2012), which reduces the communication distance between functional groups, allowing faster exchange of metabolites such as nitrite and nitrate. It implies that efficient interactions promote the accumulation of total nitrogen and conversion of NH₄^+^-N. Moreover, average degree positively correlated with NO₃^−^-N, but negative for NH₄^+^-N. A higher degree means that a species directly interacted with more species, and higher average degree represents higher network complexity (Deng et al., 2012). This result revealed that tight interactions among microbial species may stimulate conversion of NH₄^+^-N and retention of NO₃^−^-N.

Furthermore, at thermophilic phase, we found that addition of cow dung increased the number of vertices and edges, but decreased average clustering coefficient and centralization. These findings indicate that cow dung addition may introduce exogenous thermophilic bacteria in microbial network, but the elevated temperature suppresses heat-labile taxa and reduces functional connectivity and network modularity during the thermophilic phase. At cooling phase, microbial network of RHCD has higher average clustering coefficient and modularity than that of RH. As temperature decreases, diverse species introduced by cow dung may reorganize functional modules to collaboratively decompose organic matter (Zhou et al., 2010).

### 4.3 Turnover of keystone species as composting progressing

The key microbial species reveals the composting phases and corresponding microbial function. For instance, Firmicutes involved in lignocellulose degradation were dominant during the heating and thermophilic phase, while Actinobacteria involved in degradation of persistent compound was dominant at cooling phase and mature phase (Bello et al., 2020). The succession from Firmicutes to Actinobacteria indicates a functional relay: early Firmicutes dominance drives the degradation of organic matter, while Actinobacteria contribute to organic matter stabilization and pathogen suppression in later stages. Consistently, the abundance of *Actinobacteria* serving as module hubs increased in RHCD, although their overall number in the network decreased (Supplementary Figure S5). This suggests that certain *Actinobacteria*, particularly *Thermopolyspora*, became more important in structuring the microbial network. Rare taxa of *Nitriliruptoria* became connectors during the thermophilic phase, participating in nitrate reduction and both dissimilatory and assimilatory nitrogen processes (Pellegrinetti et al., 2024), which may contribute to nitrogen transformation.

Meanwhile, addition of cow dung changed the species pool of key topological roles and their links with carbon and nitrogen dynamics. For example, *Longimicrobia* specially became connector in RHCD group at the heating, thermophilic and mature phase, which also was biomarker in the digestate composting (Lu et al., 2021) and relevant with uncoated urea degradation (Bian et al., 2022). During mature phase in RHCD, *Longimicrobia* primarily formed positive interactions with key species of nitrogen transformation (Supplementary Figure S6), such as *Thermopolyspora* (capable of dissimilatory nitrate reduction to ammonium) (Wang and Li, 2023), *Filomicrobium* (capable of urea degradation and nitrate reduction) (Wu et al., 2009), and *Methanosarcina*. Among them, *Methanosarcina* also was specific connector in RHCD group at the thermophilic and mature phase, which was key methane producer and inhibited by high concentration of NH₄^+^-N (Romero-Yahuitl et al., 2024). These results revealed that microbial community reorganized a nitrogen transformation module centered on urea degradation during co-composting of rice husk and cow dung.

### 4.4 Potential mechanisms in reduction of nitrogen loss during cow dung composting

Controlling nitrogen loss during composting is an unavoidable challenge to develop peat-free seedling substrate. Physical additives promote nitrogen retention in composting process by various mechanisms, which may enhance performance of seedling substrate in lock nitrogen fertilizer during seedling. The majority of physical additives have porous characteristics and great adsorption capacity to NH_3_/NH_4_^+^, such as biochar (Chen et al., 2020), apple pomace (Jiang et al., 2014) and spent mushroom substrate (Liu et al., 2020). Also, we found that addition of cow dung increased nitrogen content in mature compost by suppressing microbial nitrogen metabolism, including denitrification processes, and reduced nitrogen loss by avoiding N_2_O emission.

Firstly, addition of cow dung increased temperature to a range from 43.8 °C to 48.2 °C after cooling phase, which was higher than the optimal temperature of denitrifying microbes (25 °C to 35 °C) and greatly restricted enzyme activities in denitrification (Braker et al., 2010). Elevated temperatures also shift the community composition by favoring thermophiles, thereby reducing the ecological niches available for denitrifiers. Secondly, additions of cow dung disturbed network structure and decreased denitrification efficiency. There were complex interaction networks for all of heterotrophic denitrification, synergetic denitrification and autotrophic denitrification (Zhang D. et al., 2023). Cooperation among intra-phylum denitrifiers could enhance denitrification efficiency, while inter-phylum cooperation among heterotrophs to provide stable environment for denitrification (Yuan et al., 2021). It implies that cow dung may disturb the interactions between denitrifiers and other species. Finally, the addition of cow dung altered the carbon pool composition and suppressed denitrification metabolism. Carbon source provides electron donor for nitrate reduction and energy for denitrification and C/N ratio played vital roles in nitrogen dynamic (Chen and Strous, 2013; Ruan et al., 2020; Yang et al., 2020). Our results showed that addition of cow dung decreased C/N ratio from 62.9 to 40.6, which may inhibit gene expression of denitrification metabolism, including *nirS*, *norB* and *nosZ* (Zhang M. et al., 2023). It was supported that carbon content has negative correlations with abundance of *nirK*, *nirS* and *norC* (Supplementary Figure S3).

## 5 Conclusion

In conclusion, this study revealed that addition of cow dung accelerated the heating rate and carbon degradation rate during rice husk composting. Cow dung could serve as buffer maintaining neutral pH throughout the whole composting process, and promoted the development of antagonistic microorganisms against plant pathogens during the cooling and mature phases, such as Actinobacteria. Moreover, we found that nitrogen dynamics during RH composting were strongly influenced by microbial interaction network pattern, but this influence was weakened after addition of cow dung. Further, our results showed that cow dung also suppressed the abundance of most denitrification-related genes at thermophilic phase, such as *norB* and *nirB*, implying the reduction of nitrogen loss in NH_3_ and N_2_O. Seedling substrates derived from both of RH and RHCD composting products exhibited higher germination rate of Solanaceae crops by 11% than commercial peat-based substrates. Germination rate and plant biomass were mainly increased by dissolved organic carbon and ammonium nitrogen in the compost products, respectively. Taken together, cow dung could accelerate the maturity of rice husk composting and reduce nitrogen loss, but may cause carbon loss. These results advance our understanding of physicochemical and biological regulation mechanisms of cow dung on rice husk composting.

## Data Availability

The datasets presented in this study can be found in online repositories. The names of the repository/repositories and accession number(s) can be found in the article/Supplementary material.

## Footnotes

^1^ https://github.com/Hy4m/linkET
