# Supplementary material for: Additive of cow dung weakened the influences of microbial interactions on nitrogen dynamic during composting of rice husks
Source: Front Microbiol. 2025 Nov 4;16:1641502. doi: 10.3389/fmicb.2025.1641502 (PMC12623384; doi:10.3389/fmicb.2025.1641502)
Supplement: Supplementary file 1 [file Data_Sheet_1.docx]

# Supplementary Information for

Cow dung additive weakened the influences of microbial interactions on nitrogen dynamic during composting of rice husks

Bin Zhang^1†^, Delong Meng^2†^, Xichun Wang^3†^, Jin Hu^2^, Jianqiang Fan^4^, Xuan Li^5^, Zhendong Yang^6^, Wei He^4^, Deying Zhou^3^, Yiqiang Cheng^4^, Jingjing Li^4^, Junliang Zou^7^, Zhenghua Liu^2*^

^1^College of Chemistry and Bioengineering, Hunan University of Science and Engineering, Yongzhou 425199, China

^2^School of Minerals Processing and Bioengineering, Central South University, Changsha 410083, China

^3^Yongzhou Company of Hunan Tobacco Company, Yongzhou 425000, China

^4^ Technology Center, Fujian Tobacco Industrial Corporation, Xiamen 361022, China

^5^College of Plant Protection, Hunan Agricultural University, Changsha 410128, China

^6^School of Architecture and Civil Engineering, Chengdu University, Chengdu 610106, China

^7^Forestry Development Department and Climate Centre, Teagasc, Johnstown Castle, Y35 TC97, Wexford, Ireland

†These authors contributed equally to this work.

^*^Corresponding author. E-mail: Zhenghua Liu, [liuzhenghua2017csu@163.com](mailto:liuzhenghua2017csu@163.com)

**The file includes:**

Supplementary Tables (S1–S4)

Supplementary Figures (S1–S6)

Table S1. The content of carbon and nitrogen of composting materials

| Materials | Total carbon (%) | Total nitrogen (%) | C:N |
| --- | --- | --- | --- |
| Rice husk | 46.40 | 0.48 | 75.00 |
| Cow dung | 35.30 | 2.06 | 18.93 |
| Urea | 20.20 | 46.60 | 0.43 |
| Corn starch | 46.70 | 0.48 | 97.29 |
| Fermentation aids | 41.20 | 2.08 | 19.81 |

Table S2. The composition of each substrate is presented in the table below (volume ratio, %)

| Group | Rice husk composting | Rice husk-cow dung composting | Coconut Coir | Perlite | Peat | Carbonized rice husk |
| --- | --- | --- | --- | --- | --- | --- |
| RH | 45% | – | 35% | 20% | – | – |
| RHCD | - | 45% | 35% | 20% | – | – |
| PSS | – | – | – | 27% | 32% | 41% |

Table S3. The fitting effects of linear regression model for network properties and environmental factor

| Group | Network properties | Environmental factor | *P* | *R*^2^ |
| --- | --- | --- | --- | --- |
| RH | Vertex | pH | **0.010** | **0.349** |
| RH | Edge | pH | **0.006** | **0.384** |
| RH | Average degree | pH | **0.004** | **0.422** |
| RH | Average path length | pH | **0.008** | **0.368** |
| RH | Density | pH | 0.088 | 0.171 |
| RHCD | Vertex | pH | 0.797 | 0.004 |
| RHCD | Edge | pH | 0.996 | 0.000 |
| RHCD | Average degree | pH | 0.878 | 0.002 |
| RHCD | Average path length | pH | 0.591 | 0.018 |
| RHCD | Density | pH | 0.468 | 0.033 |
| RH | Vertex | TC | **0.001** | **0.519** |
| RH | Edge | TC | **0.001** | **0.485** |
| RH | Average degree | TC | **0.002** | **0.456** |
| RH | Average path length | TC | **0.010** | **0.350** |
| RH | Density | TC | **0.002** | **0.463** |
| RHCD | Vertex | TC | 0.152 | 0.124 |
| RHCD | Edge | TC | 0.151 | 0.125 |
| RHCD | Average degree | TC | 0.125 | 0.141 |
| RHCD | Average path length | TC | 0.180 | 0.110 |
| RHCD | Density | TC | 0.294 | 0.069 |
| RH | Vertex | TN | 0.086 | 0.173 |
| RH | Edge | TN | 0.147 | 0.127 |
| RH | Average degree | TN | 0.085 | 0.174 |
| RH | Average path length | TN | **0.016** | **0.311** |
| RH | Density | TN | **0.046** | **0.227** |
| RHCD | Vertex | TN | 0.142 | 0.130 |
| RHCD | Edge | TN | 0.117 | 0.146 |
| RHCD | Average degree | TN | 0.124 | 0.141 |
| RHCD | Average path length | TN | 0.175 | 0.112 |
| RHCD | Density | TN | 0.410 | 0.043 |
| RH | Vertex | NH_4_^+^-N | **0.002** | **0.466** |
| RH | Edge | NH_4_^+^-N | **0.004** | **0.413** |
| RH | Average degree | NH_4_^+^-N | **0.002** | **0.467** |
| RH | Average path length | NH_4_^+^-N | **0.004** | **0.412** |
| RH | Density | NH_4_^+^-N | **0.001** | **0.491** |
| RHCD | Vertex | NH_4_^+^-N | **0.024** | **0.278** |
| RHCD | Edge | NH_4_^+^-N | **0.031** | **0.259** |
| RHCD | Average degree | NH_4_^+^-N | **0.018** | **0.301** |
| RHCD | Average path length | NH_4_^+^-N | **0.036** | **0.247** |
| RHCD | Density | NH_4_^+^-N | **0.041** | **0.235** |
| RH | Vertex | NO_3_^-^-N | **0.007** | **0.372** |
| RH | Edge | NO_3_^-^-N | **0.004** | **0.421** |
| RH | Average degree | NO_3_^-^-N | **0.006** | **0.380** |
| RH | Average path length | NO_3_^-^-N | **0.030** | **0.263** |
| RH | Density | NO_3_^-^-N | **0.045** | **0.228** |
| RHCD | Vertex | NO_3_^-^-N | 0.677 | 0.011 |
| RHCD | Edge | NO_3_^-^-N | 0.867 | 0.002 |
| RHCD | Average degree | NO_3_^-^-N | 0.707 | 0.009 |
| RHCD | Average path length | NO_3_^-^-N | 0.392 | 0.046 |
| RHCD | Density | NO_3_^-^-N | 0.428 | 0.040 |

Table S4. Relationships among the variables in partial least squares path model

| Group | Relationships | Direct | Indirect | Total |
| --- | --- | --- | --- | --- |
| RH | NetSize -> NetComp | -0.9522 | 0.0000 | -0.9522 |
|  | NetSize -> Cpool | -1.2990 | 0.5196 | -0.7794 |
|  | NetSize -> NG1 | -0.2326 | 1.0099 | 0.7773 |
|  | NetSize -> NG2 | 0.7599 | -1.2479 | -0.4880 |
|  | NetSize -> Npool1 | 0.0815 | 0.6008 | 0.6824 |
|  | NetSize -> Npool2 | 3.2320 | -2.6345 | 0.5975 |
|  | NetComp -> Cpool | -0.5457 | 0.0000 | -0.5457 |
|  | NetComp -> NG1 | -1.0606 | 0.0000 | -1.0606 |
|  | NetComp -> NG2 | 1.3106 | 0.0000 | 1.3106 |
|  | NetComp -> Npool1 | 0.2656 | -1.0844 | -0.8188 |
|  | NetComp -> Npool2 | 2.5215 | -1.2809 | 1.2406 |
|  | Cpool -> Npool1 | -0.0474 | 0.0000 | -0.0474 |
|  | Cpool -> Npool2 | 0.8042 | 0.0000 | 0.8042 |
|  | NG1 -> Npool1 | 1.0548 | 0.0000 | 1.0548 |
|  | NG1 -> Npool2 | 0.2084 | 0.0000 | 0.2084 |
|  | NG2 -> Npool1 | 0.0064 | 0.0000 | 0.0064 |
|  | NG2 -> Npool2 | -0.4739 | 0.0000 | -0.4739 |
| RHCD | NetSize -> NetComp | -0.9624 | 0.0000 | -0.9624 |
|  | NetSize -> Cpool | -0.9812 | 0.5694 | -0.4118 |
|  | NetSize -> NG1 | 0.3156 | 0.3918 | 0.7073 |
|  | NetSize -> NG2 | -0.3602 | 0.2107 | -0.1496 |
|  | NetSize -> Npool1 | -0.1557 | 0.6807 | 0.5249 |
|  | NetSize -> Npool2 | 1.5994 | -1.6828 | -0.0834 |
|  | NetComp -> Cpool | -0.5917 | 0.0000 | -0.5917 |
|  | NetComp -> NG1 | -0.4071 | 0.0000 | -0.4071 |
|  | NetComp -> NG2 | -0.2189 | 0.0000 | -0.2189 |
|  | NetComp -> Npool1 | -0.1110 | -0.0233 | -0.1344 |
|  | NetComp -> Npool2 | 1.7700 | -0.4784 | 1.2916 |
|  | Cpool -> Npool1 | -0.3227 | 0.0000 | -0.3227 |
|  | Cpool -> Npool2 | 0.7600 | 0.0000 | 0.7600 |
|  | NG1 -> Npool1 | 0.5960 | 0.0000 | 0.5960 |
|  | NG1 -> Npool2 | 0.3584 | 0.0000 | 0.3584 |
|  | NG2 -> Npool1 | -0.1294 | 0.0000 | -0.1294 |
|  | NG2 -> Npool2 | -0.5356 | 0.0000 | -0.5356 |


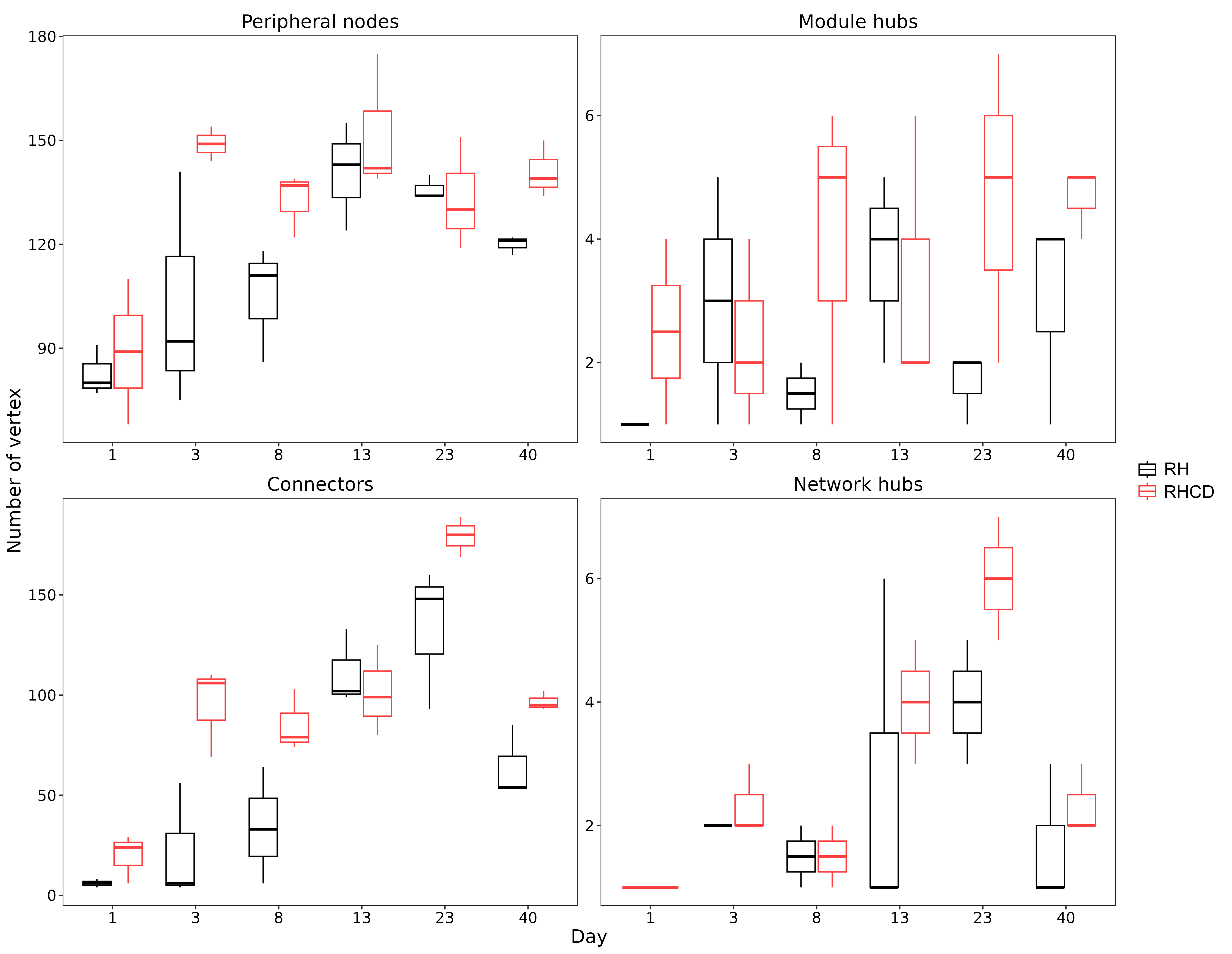


Figure S1. Changes in number of network topological roles during composting process.


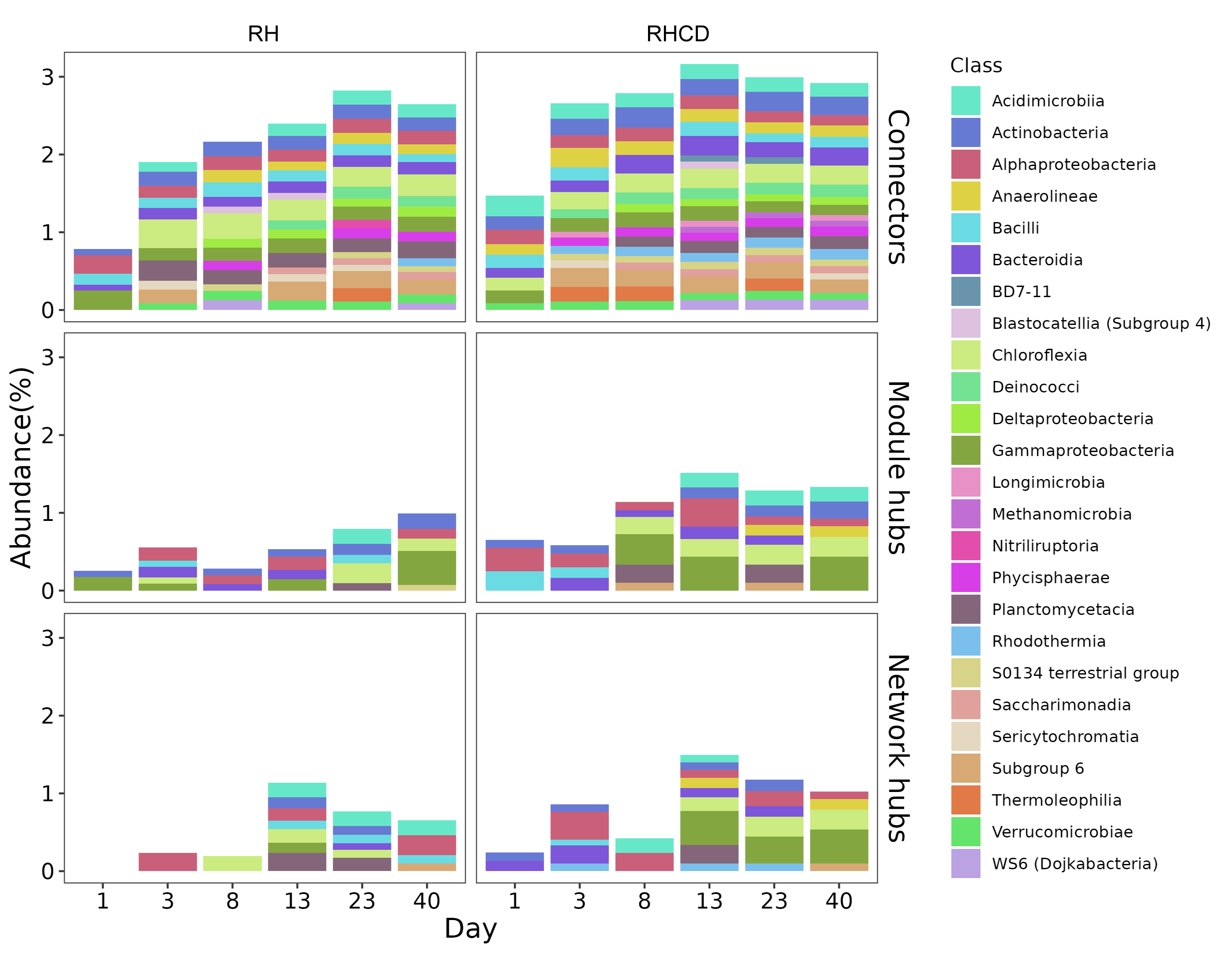


Figure S2. Species composition of network topological roles at class level.


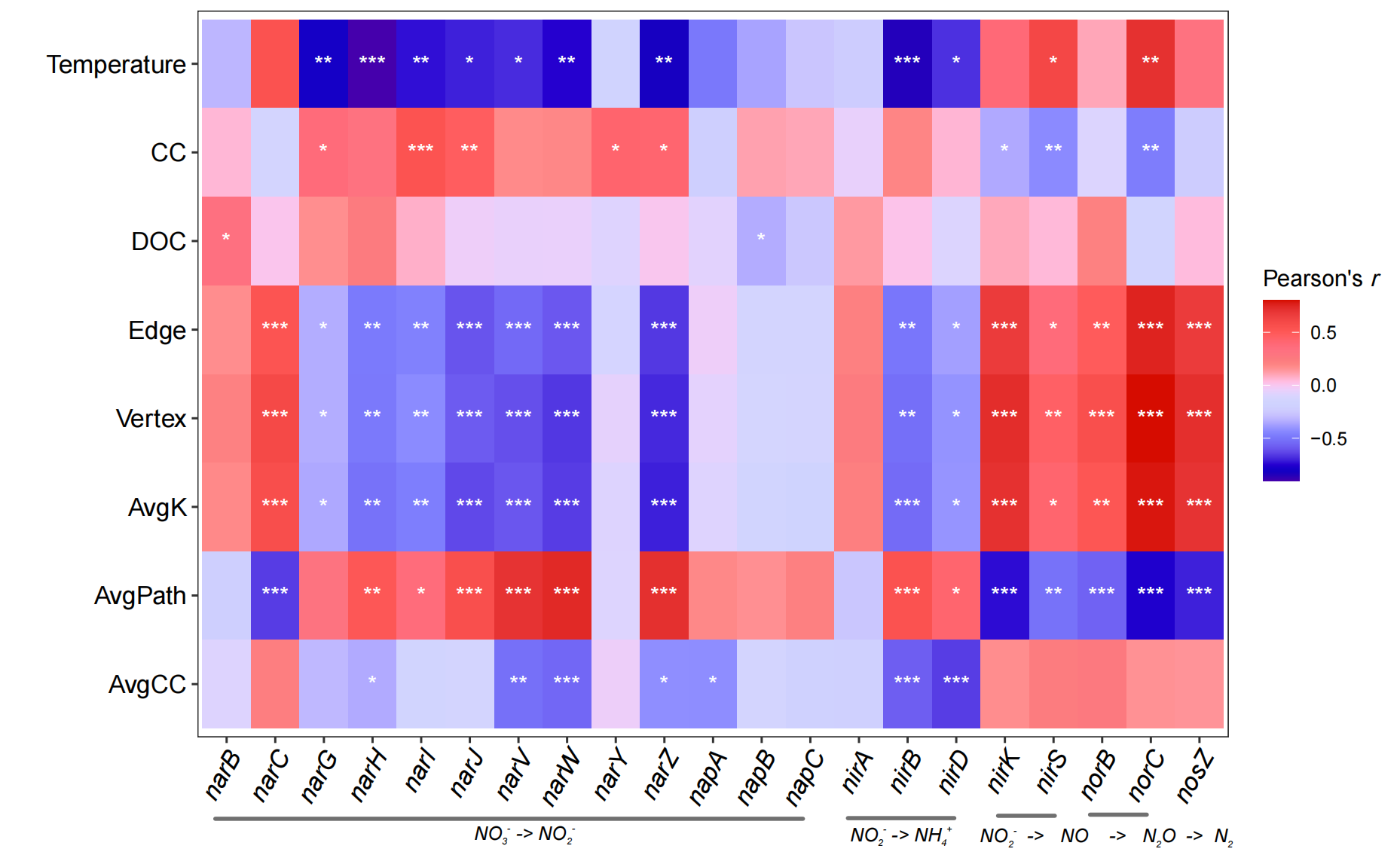


Figure S3. Correlations between denitrification genes and temperature, carbon content and network structure. All *P* < 0.05.


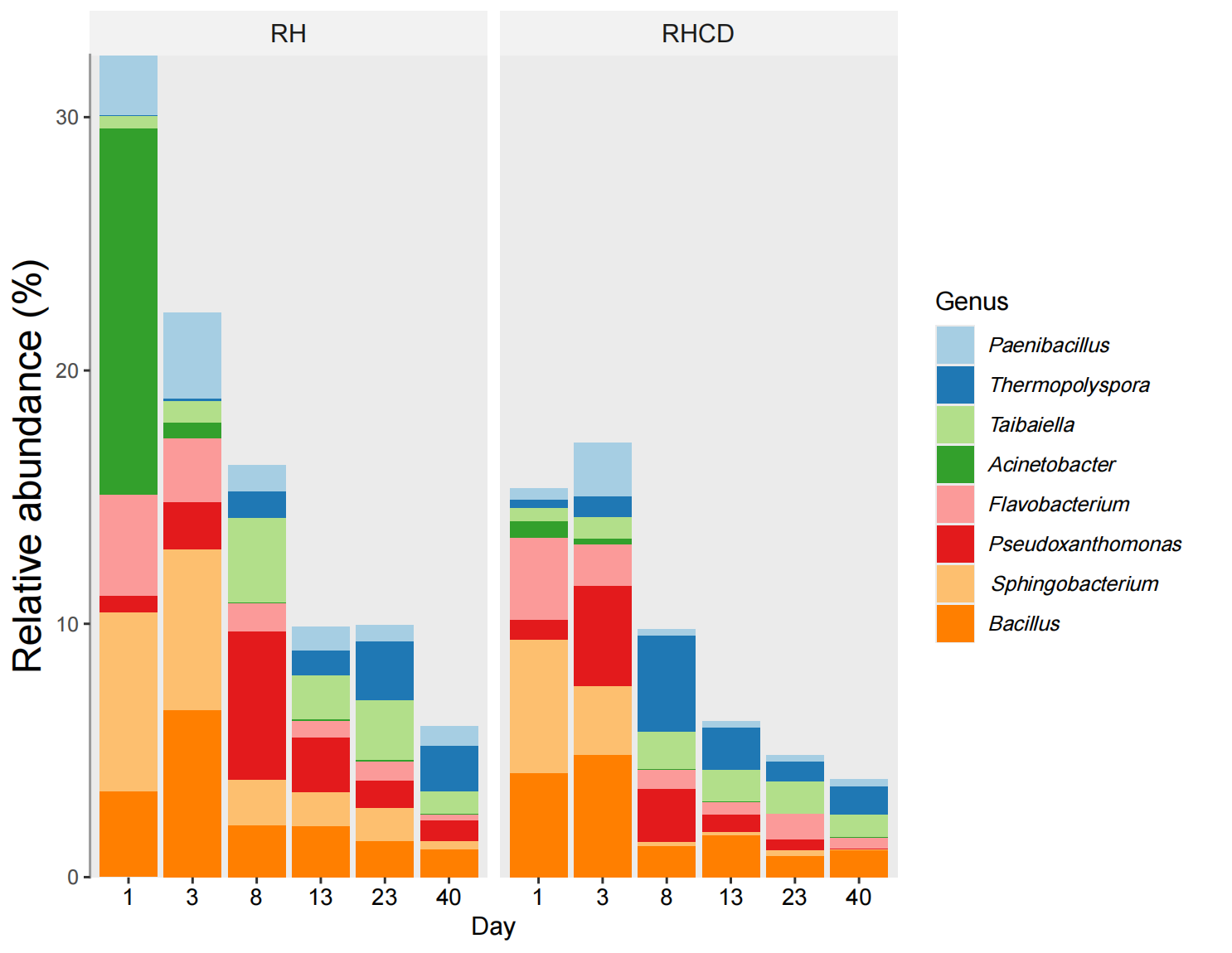


Figure S4. Relative abundance of dominant taxa at genus level.


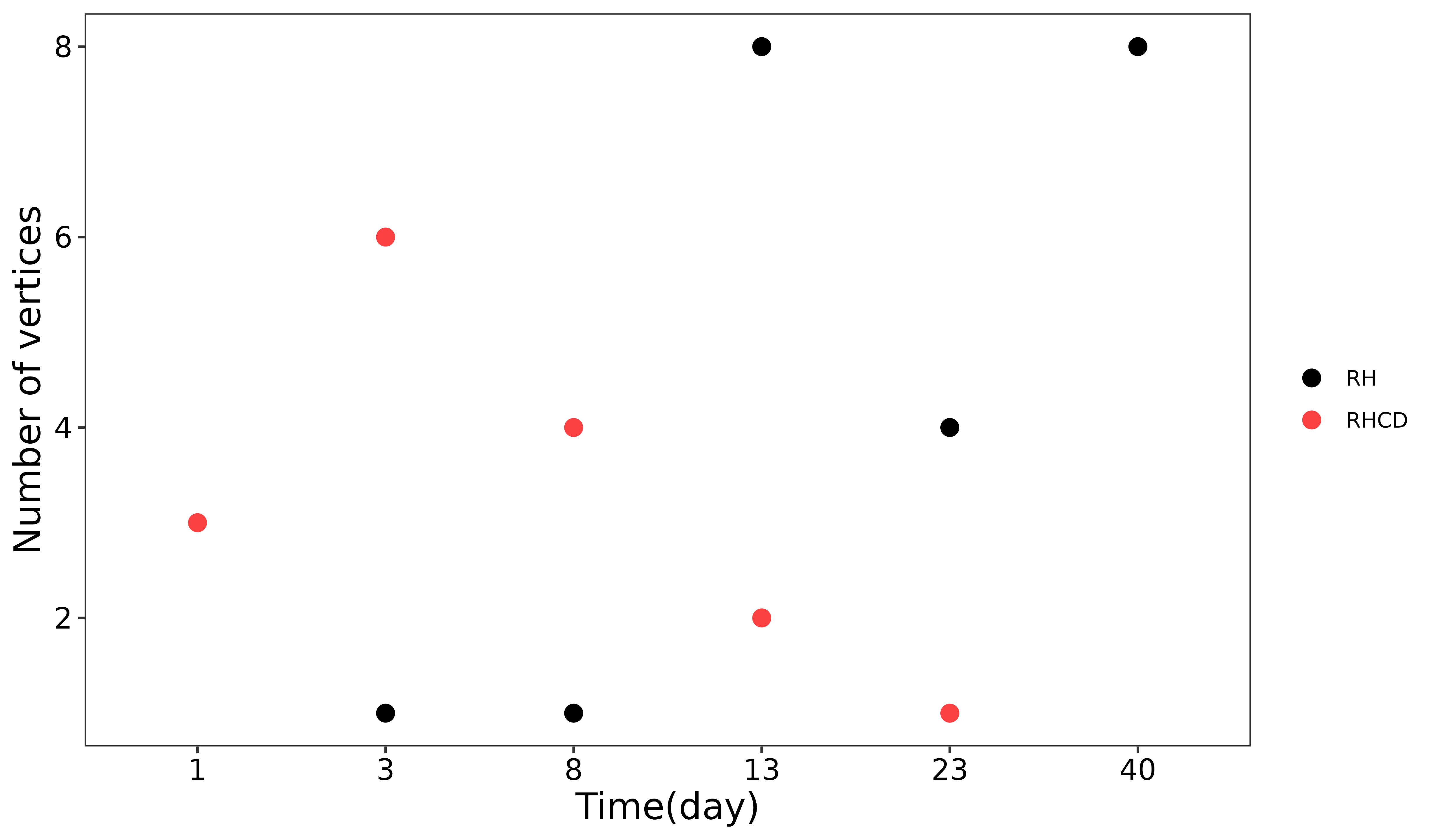


Figure S5. Number of vertices represented by Actinobacteria species as module hub in microbial network.


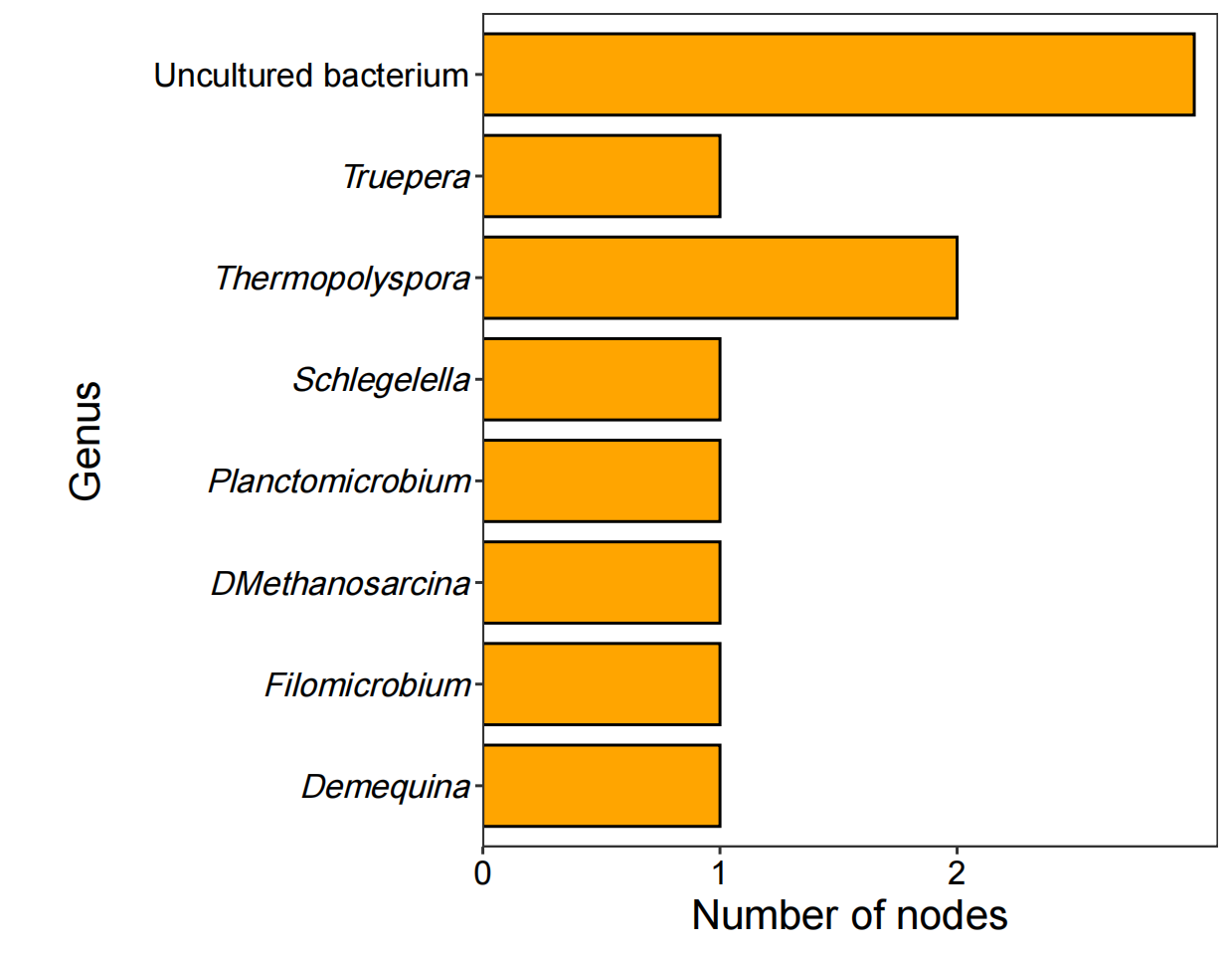


Figure S6. Neighbors of Longimicrobia in microbial network in RHCD at mature phase.
